# Physical Exercise Improves Symptomatic Dermographism

**DOI:** 10.1002/clt2.70083

**Published:** 2025-07-23

**Authors:** Ragıp Ertaş, Muhammed Burak Yücel, Murat Türk, Şule Ketenci Ertaş, Emek Kocatürk, Melba Muñoz

**Affiliations:** ^1^ Urticaria Center of Reference and Excellence (UCARE) Chronic Skin Diseases Unit Department of Dermatology University of Health Sciences Kayseri City Education and Research Hospital Kayseri Turkey; ^2^ Division of Allergy and Clinical Immunology Urticaria Center of Reference and Excellence (UCARE) Faculty of Medicine Erciyes University Kayseri Turkey; ^3^ Department of Internal Medicine Division of Rheumatology Kayseri Acıbadem Hospital Kayseri Turkey; ^4^ Department of Dermatology Bahçeşehir University School of Medicine İstanbul Turkey; ^5^ Institute of Allergology, Charité—Universitätsmedizin Berlin, Corporate Member of Freie Universität Berlin and Humboldt‐Universität zu Berlin Berlin Germany; ^6^ Fraunhofer Institute for Translational Medicine and Pharmacology ITMP, Immunology and Allergology Berlin Germany

**Keywords:** chronic urticaria, exercise, physical activity, symptomatic dermographism

## Abstract

**Background:**

Short‐term exercise may reduce disease activity in symptomatic dermographism (SD), but its prevalence and short‐ and long‐term effects remain unclear and understudied. This study aims to assess the impact of both short‐term and regular long‐term exercise programs on disease activity in patients with SD.

**Methods:**

We performed a short‐term exercise test to assess the disease activity and the critical friction threshold (CFT) using the FricTest before (SDE1) and 10 min after (SDE2) this test on 34 SD patients. Afterward, we asked the patients to carry on a 1‐month regular long‐term exercise program according to the World Health Organization's physical activity recommendations. At the end of this 1‐month period, we performed the short‐term exercise test using the FricTest before (SDE3) and 10 min after (SDE4) the exercise test.

**Results:**

Before a 1‐month regular exercise program, 32 of 34 patients (94.1%) showed a reduction in the critical friction threshold after the short‐term exercise test (SDE1; 1.95 ± 0.88 vs. SDE2; 0.81 ± 0.86). After a 1‐month regular exercise program, 29 of 34 patients (85%) showed a reduction in SD symptoms with short‐term exercise test and the FricTest scores were significantly decreased (SDE3; 1.57 ± 0.80 vs. SDE4; 1.01 ± 0.83). After the 1‐month regular exercise program, a statistically significant increase was seen in the patients’ UCT scores and quality of life.

**Conclusions:**

Our findings show that short‐term exercise improves SD symptoms, while long‐term regular exercise programs reduce disease symptoms and improve UCT scores and quality of life.

**Abbreviations**
ACARE: angioedema center of reference and excellence
CIndU: chronic inducible urticaria
CSDU: chronic skin diseases unit
CSU: chronic spontaneous urticaria
CU: chronic urticaria
HPA: hypothalamic–pituitary–adrenal axis
IQR: interquartile range
SD: symptomatic dermographism
SDE: symptomatic dermographism score
UAS: urticaria activity score
UCARE: urticaria center of reference and excellence
UCT: urticaria control test

## 1 Introduction

Symptomatic Dermographism (SD) is the most common subtype of chronic inducible urticaria (CIndU), characterized by the development of recurrent itchy wheals, and sometimes accompanying CSU [1] and/or angioedema [2] after stroking, rubbing or scratching the skin. These reactions can last up to 30 min [3]. SD usually affects young adults, and its prevalence is estimated to be 2%–5% [3]. Patients with chronic urticaria display a marked quality of life impairment that can also affect their sexual activity. Likewise, 44% of patients with SD have reported deterioration of their quality of life [4, 5].

The diagnosis of SD is mainly based on provocation tests and detailed patient clinical history. Skin provocation test responses obtained using the FricTest, correlate with disease activity, quality of life, pruritus score, and the clinical course of disease in patients with SD [6].

Second generation antihistamines (sgAH) and an off‐label therapy with omalizumab can modify trigger thresholds using the FricTest and decrease disease activity in patients with SD [7]. However, additional factors that affect trigger thresholds and disease activity are not known [8]. We have recently identified two novel variants in SD patients, food‐exacerbated SD (FE‐SD) and food‐dependent SD (FD‐SD) [9]. In this study, food intake induced positive whealing responses to provocation testing in some patients who did not have SD in the past (FD‐SD). The other and larger proportion of SD patients showed increased disease activity and lower postprandial whealing responses after provocation test (FE‐SD). We also recently found that after food intake disease activity increased in a pediatric patient group [10]. Furthermore, we have also demonstrated that exercise can reduce disease activity and decrease whealing responses after provocation testing [11].

In this study, we aimed to assess the impact of short‐ and long‐term exercise on disease activity and trigger thresholds after provocation testing in patients with SD.

## 2 Materials and Methods

### 2.1 Study Conduct and Patients

A total of 34 patients treated at our chronic skin diseases Unit (CSDU [12]), urticaria center of reference and excellence (UCARE [13]), and angioedema center of reference and excellence (ACARE [14]) between January 2022 and June 2022 were prospectively included in the study. All approvals for the study were obtained with the decision numbered 452 and dated July 29, 2021 from the Ethics Committee of the University of Health Sciences, Kayseri City Training and Research Hospital. All participants provided written consent.

### 2.2 Assessment of Whealing Responses to Provocation With FricTest

Skin provocation testing responses with FricTest (Moxie, Berlin, Germany) were assessed in 34 SD patients [15]. The FricTest is a comb‐like instrument with 4 pins (3.0, 3.5, 4.0, and 4.5 mm) used to diagnose SD that determines trigger thresholds, that is the shortest pin length/minimum pressure that elicits an itchy wheal response. The FricTest was performed on the volar forearm while patients were at rest, not taking additional medications and had not used sgAH for at least 3 days.

All tests were conducted by the same researcher and at the same times of the day (between 10:00 a.m. and 12:00 a.m.). Responses were recorded at 12 time points after the use of FricTest, that is every minute up to 10 min as well as 5 and 30 s after testing. All responses were recorded as negative (scored as −) or positive (scored from + to ++++ according to the trigger value producing an itchy wheals). Total FricTest scores at 12 time points were summed and divided by 12 to obtain the patient’s FricTest score. The higher a patient’s FricTest score, the greater the SD disease activity.

### 2.3 Assessment of Short‐Term Exercise Test and Long‐Term Regular Exercise Program on Wheal Responses to Provocation Testing

In order to determine the short‐term effects of exercising on CFT, all patients fasted, until basal SDE values (SDE1) were assessed, short‐term exercise was performed and SDE values (SDE2) were evaluated again 10 min after exercise. Short‐term exercise, which means walking up and down a 10‐step staircase 10 times or walking on a treadmill at 6 km/h for 10 min to increase heart rate to 140–170 beats per minute.

In order to identify the long‐term effect of exercise, a 1‐month moderate‐intensity exercise program was recommended to the patients. Patients were asked to exercise daily (long‐term regular exercise) for 30 days, by performing at least 30 min of moderate‐intensity exercise per day (in line with the WHO recommendation of 150 min per week), using their smartphones or smart watches.

**FIGURE 1:**
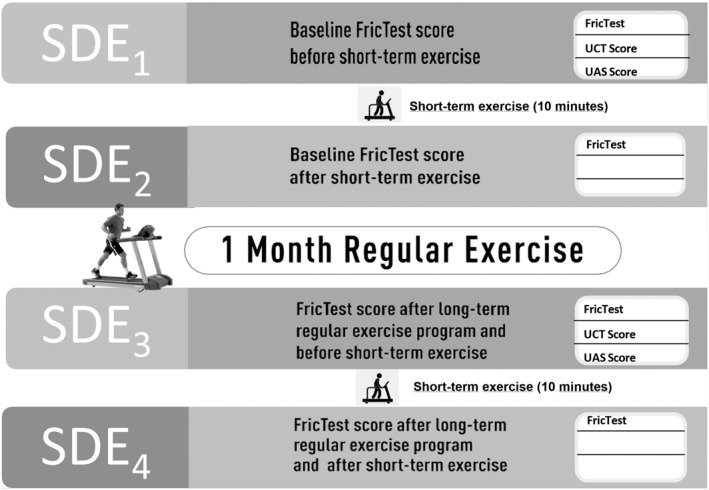
Summary of study protocol. FricTest scores were evaluated at before (SDE1) and 10 min after (SDE2) short‐term exercise, and before (SDE3) and 10 min after (SDE4) short‐term exercise following 1 month long‐term exercise. Additionally, urticaria control test (UCT) and in‐clinic urticaria activity score (UAS) was assessed both before and after the long‐term exercise program.

The patients’ general activities consisted of irregular steps taken while commuting to work and at home. That means, none of our patients had a history of regular exercise. At the end of 1 month, patients fasted before CFT were assessed (SDE3) and then they performed a short‐term exercise test again and CFT (SDE4) were evaluated again 10 min after the provocation test (Figure 1).

The total number of steps of the patients in a 1‐month period was also evaluated with the PedoMeter smart phone and the smart watch integrated application, divided by 30 to determine the daily step number. According to the number of daily steps, the patients were divided into three categories as below 4000 steps, between 4000 and 8000 steps and above 8000 steps. In addition to the number of steps, we also determined whether the patients exercised regularly or not. For this purpose, we divided the patients into two groups as patient with regular or irregular exercise. Patients who exercise regularly, performed a physical activity every day without interruption and patients who exercise irregularly did not follow the exercise program regularly and took a break for 1 day or more.

### 2.4 Assessment of Disease Activity and Control of Comorbid CSU

To assess CSU disease activity, 29 of 34 SD patients who had comorbid chronic spontaneous urticaria (CSU) filled out the in‐clinic urticaria activity score (UAS). Patients were asked about the daily number of wheals (no wheals = 0 points; < 20 wheals = 1 point; 20–50 wheals = 2 points; > 50 wheals = 3 points) and the intensity of itch (no itch = 0 points; mild itch = 1 point; moderate itch = 2 points; severe itch = 3 points) the day before of the test.

Urticaria control test (UCT, Moxie, Berlin, Germany) was used to assess disease control in all patients [16]. UCT consists of 4 questions with 5 answer options each. Each answer is given a score between 0 and 4. Afterward, scores of all 4 questions are added together. Accordingly, the minimum and maximum UCT scores are 0 and 16, respectively. A score of 16 indicates complete disease control, whereas a score below 12 indicates poorly controlled disease.

All included patients continued their treatment without any change during the month they exercised. Of the 34 patients, 22 were treated with a standard‐dosed sgAH, 3 with high dosed sgAH, 6 with omalizumab monotherapy, and 3 patients used omalizumab and high‐dosed sgAH.

### 2.5 Statistical Analysis

Kolmogorov‐Smirnov test was used to specify the variables distribution. Parametric and nonparametric variables were evaluated with normality tests and histogram. Parametric values were analyzed using mean ± standard deviation (SD), while non‐parametric values were analyzed as median (interquartile ranges; IQR). Categorical variables were defined using the chi‐square test. Paired‐T‐Test was used to evaluate the two dependent variable groups. Wilcoxon Test was used to evaluate 2 groups of non‐parametric dependent variables. *p* < 0.05 was used to determine statistical significance.

## 3 Results

### 3.1 In Patients With SD, Short‐Term Exercise Testing Reduces Whealing Responses to Provocation Testing

**FIGURE 2:**
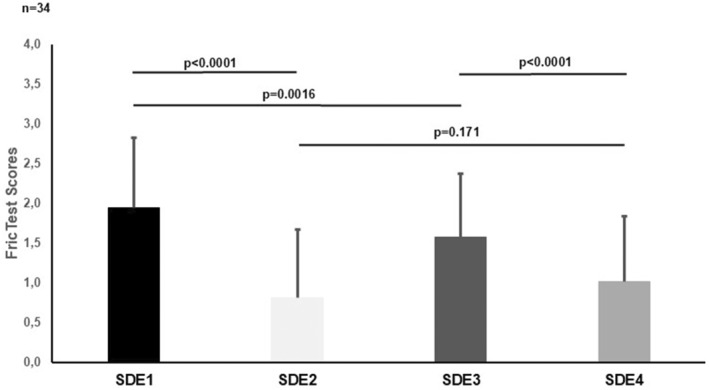
General characteristics of the study according to FricTest scores. SDE_1_: Baseline FricTest score before short‐term exercise. SDE_2_: Baseline FricTest score after short‐term exercise. SDE_3_: FricTest score after long‐term regular exercise program and before short‐term exercise. SDE_4_: FricTest score after long‐term regular exercise program and after short‐term exercise. *Paired *t*‐Test.

Of 34 SD patients with a mean age of 38.7 ± 11.7 years, 24 (70.6%) were female. At the beginning of the study prior to the regular exercise program, 32 of 34 patients (94.1%) had reduced CFT after the short‐term exercise testing reducing FricTest scores from 1.95 ± 0.88 (SDE1) to 0.81 ± 0.86 (SDE2) (*p* < 0.0001). The impact of a short‐term exercise test was examined again after a one‐month‐regular exercise program. FricTest scores were also reduced from 1.57 ± 0.80 (SDE3) to 1.01 ± 0.83 (SDE4; *p* < 0.0001, Table 1, Figure 2). Individual FricTest scores for all patients at each measurement point (SDE1, SDE2, SDE3, and SDE4) are presented in Supporting Information S1: Table 1, highlighting the consistency of exercise‐induced changes in whealing responses.

**TABLE 1:** Results of the study.

All patients (*n*:34)	Mean ± SD	*p*
SDE_1_	1.95 ± 0.88	**<** **0.0001** ^a^
SDE_2_	0.81 ± 0.86	
After 1 month of regular exercise program		
SDE_3_	1.57 ± 0.80	**<** **0.0001** ^a^
SDE_4_	1.01 ± 0.83	
SDE_1_	1.95 ± 0.88	**0.016** ^a^
SDE_3_	1.57 ± 0.80	
SDE_2_	0.81 ± 0.86	0.171^a^
SDE_4_	1.01 ± 0.83	
BEFORE long‐term exercise program ΔSDE1–SDE2; % (IQR)	−70 [(−100) ‐ (−37.35)]	**0.003** ^b^
AFTER long‐term exercise program ΔSDE3–SDE4; % (IQR)	−40.4 [(−63.1) ‐ (−9.56)]	
Baseline in‐clinic UAS (*n*:29)	1.56 ± 1.71	0.062^a^
Control in‐clinic UAS (*n*: 29)	0.88 ± 1.20	
UCT0	11.7 ± 3.5	**0.043** ^a^
UCT1	13 ± 2.4	

*Note:* Baseline in‐clinic UAS: Urticaria activity score before the long‐term regular exercise program. Control in‐clinic UAS: Urticaria activity score after long‐term regular exercise program. SDE_1_: Baseline FricTest score before short‐term exercise. SDE_2_: Baseline FricTest score after short‐term exercise. SDE_3_: FricTest score after long‐term regular exercise program and before short‐term exercise. SDE_4_: FricTest score after long‐term regular exercise program and after short‐term exercise. UCT0: Urticaria control test score before the long‐term regular exercise program. UCT1: Urticaria control test score after the long‐term regular exercise program. *p*‐values in bold are statistically significant.

^a^ Paired *t*‐test.

^b^ Wilcoxon test.

### 3.2 In Patients With SD, Long‐Term Regular Exercise During One Month Reduces Whealing Responses to Provocation Testing

After 1 month of regular physical exercise, whealing responses to FricTest provocation were significantly decreased by approximately 20% when compared to the baseline scores (SDE1: 1.95 ± 0.88 and SDE3: 1.57 ± 0.8; *p* = 0.016; Table 1, Figure 2).

High intensity and regular physical exercise during 1 month were linked to greater reduction in trigger‐induced whealing responses. Exercising at low intensity during 1 month (< 4000 steps/day, 2.44 ± 0.55[SDE1] vs. 2.26 ± 0.7[SDE3]), moderate intensity (4000–8000 steps/day, 1.78 ± 0.92[SDE1] vs. 1.38 ± 0.72[SDE3]), and high intensity (> 8000 steps/day, 1.84 ± 0.93[SDE1] vs. 1.28 ± 0.70[SDE3]) reduced FricTest scores by 0.18, 0.4, and 0.56 points, respectively (Table 2).

**TABLE 2:** FricTes**t**, UCT, and UAS scores changing according to exercise intensit**y**.

	All patients (*n* = 34)	Steps number < 4000 per day (*n* = 8)	Steps number 4000–8000 per day (*n* = 19)	Steps number > 8000 per day (*n* = 7)	*p* (*n* = 34)
SDE_1_	1.95 ± 0.88	2.44 ± 0.55	1.78 ± 0.92	1.84 ± 0.93	*p* = 0.200
SDE_2_	0.81 ± 0.86	1.00 ± 0.83	0.68 ± 0.85	0.77 ± 0.78	*p* = 0.665
SDE_3_	1.57 ± 0.80	2.26 ± 0.70	1.38 ± 0.72	1.28 ± 0.70	**p = 0.014** ^a^
SDE_4_	1.01 ± 0.83	1.56 ± 1.18	0.84 ± 0.67	0.85 ± 0.53	*p* = 0.099

*Note:* SDE_1_: Baseline FricTest score before short‐term exercise. SDE_2_: Baseline FricTest score after short‐term exercise. SDE_3_: FricTest score after long‐term regular exercise program and before short‐term exercise. SDE_4_: FricTest score after long‐term regular exercise program and after short‐term exercise. The “All patients” column represents the total cohort of 34 patients included in the study; the subsequent columns display the mean values for the respective exercise intensity and regularity subgroups. *p*‐value in bold is statistically significant.

^a^ One‐way ANOVA test.

Accordingly, the FricTest scores of the patients with low‐intensity (< 4000 steps/day) regular exercise program during 1 month were significantly higher than those of the patients with moderate (4000–8000 steps/day) and high‐intensity (< 8000 steps/day) regular exercise program (*p* = 0.014; Table 2).

Long‐term regular exercise reduced FricTest scores in patients who exercised every day, but not in those who did not (+ 0.3 vs. −0.59). In addition, patients who regularly exercised during 1 month had lower UAS scores (2.00 ± 1.60 vs. 0.53 ± 0.81; *p* = 0.001) and significantly higher UCT (11.12 ± 1.80 vs. 13.50 ± 2.35; *p* = 0.013) compared to that of patients who irregularly exercised (Table 3). However, there was no significant change in UAS and UCT scores between the groups.

**TABLE 3:** FricTest, UCT, and UAS scores changing according to exercise regularity.

	All patients (*n* = 34)	Exercise regularity (−) (*n* = 8)	Exercise regularity (+) (*n* = 26)	*p* (*n* = 34)
SDE_1_	1.95 ± 0.88	1.40 ± 0.90	2.11 ± 0.81	**p = 0.042** ^b^
SDE_2_	0.81 ± 0.86	0.76 ± 0.79	0.77 ± 0.84	*p* = 0.957
SDE_3_	1.57 ± 0.80	1.70 ± 1.10	1.52 ± 0.70	*p* = 0.579
SDE_4_	1.01 ± 0.83	1.05 ± 1.20	1.00 ± 0.70	*p* = 0.887
Before UAS4	1.56 ± 1.71	2.12 ± 1.12	1.38 ± 1.83	*p* = 0.291
After UAS4	0.88 ± 1.20	2.00 ± 1.60	0.53 ± 0.81	**p = 0.001** ^b^
UCT0	11.7 ± 3.5	10.37 ± 3.62	12.07 ± 3.48	*p* = 0.240
UCT1	13 ± 2.4	11.12 ± 1.80	13.50 ± 2.35	**p = 0.013** ^b^
Steps number < 4000 per day (*n* = 8)	8	3 (37.5%)	5 (62.5%)	*p* = 0.531^a^
Steps number 4000–8000 per day (*n* = 19)	19	4 (21%)	15 (79%)	
Steps number > 8000 per day (*n* = 7)	7	1 (14.2%)	6 (84.8%)	

*Note:* SDE_1_: Baseline FricTest score before short‐term exercise. SDE_2_: Baseline FricTest score after short‐term exercise. SDE_3_: FricTest score after long‐term regular exercise program and before short‐term exercise. SDE_4_: FricTest score after long‐term regular exercise program and after short‐term exercise. UAS: Urticaria activity score. UCT: Urticaria control test. The “All patients” column represents the total cohort of 34 patients included in the study; the subsequent columns display the mean values for the respective exercise intensity and regularity subgroups. *p*‐values in bold are statistically significant.

^a^ Chi‐square test.

^b^ One‐way ANOVA test.

### 3.3 Long‐Term Regular Exercise Decreases SD Whealing Responses to Provocation Testing After Short‐Term Exercise

At the end of 1 month of exercise, 29 of 34 (%85) patients showed a reduction in FricTest scores, 3 exhibited no change, and 2 showed increased FricTest scores. A more significant reduction in FricTest scores was found after the short‐term exercise test and before the exercise program than after 1 month of regular exercise program (Table 1; ΔSDE1–2: −70% [IQR: −100%, −37.4%] vs. ΔSDE3–4 −40.4% [−63.1%, −9.6%]; *p* = 0.003).

Before exercising for 1‐month, short‐term exercise resulted in complete and partial protection from provocation‐induced whealing in 12 and 20 patients, respectively (two patients had no benefit). After 1 month of exercise, only one patient was completely protected from provocation‐induced whealing after short‐term exercise, 29 patients showed partial protection, and 5 did not benefit.

### 3.4 In Patients With SD, One Month of Physical Exercise Reduced Disease Activity of Comorbid Chronic Spontaneous Urticaria and Improved Overall Urticaria Control

Of our 34 patients with SD, 29 had comorbid chronic spontaneous urticaria (CSU). As assessed by use of the in‐clinic UAS, 1 month of physical exercise slightly reduced disease activity of their comorbid CSU by 44%, from mean (± SD) UAS values of 1.56 ± 1.71 to 0.88 ± 1.20, albeit not statistically significantly (*p* = 0.062; Table 1). After 1 month of physical exercise urticaria control increased, as assessed by the use of the UCT, by 1.3 points (UCT baseline: 11.7 ± 3.5; UCT post exercise: 13 ± 2.4; *p* = 0.043; Table 1). Before exercising for 1 month, 15 of 34 SD patients had uncontrolled disease, that is a UCT score of less than 12. At the end of 1 month of physical exercise, 9 of these 15 patients (60%) achieved well controlled disease.

### 3.5 Long‐Term and Regular Exercise Were Linked to the Improvement of Comorbid CSU Activity and Overall Urticaria Control

After 1 month of exercise, UAS scores were lower (1.38 ± 1.83 vs. 0.53 ± 0.81, *p* = 0.001) and UCT scores were significantly higher (12.07 ± 3.48 vs. 13.5 ± 2.35, *p* = 0.013; Table 3) in patients who exercised regularly.

## 4 Discussion

Our study highlights that short‐term exercise testing reduces whealing responses in the provocation test. In addition, lower whealing responses were also observed after 1 month of regular exercise. These findings shed light on novel factors in the pathogenesis of SD and can lead to applying different methods in the treatment of SD patients. We have previously revealed that SD worsened due to food intake since SD severity and whealing responses in the provocation test increased after eating in most patient groups. Positive provocation testing was also found after food intake in some patients who did not have a previous history of SD group [9]. Furthermore, exercise decreased whealing responses in the FricTest scores and protects against eating‐induced worsening in patients with SD [11]. It is still unclear how exercise reduces SD activity and how regular exercise program influences the change in reduction.

The hypothalamic–pituitary–adrenal axis (HPA) might be involved in the modulation of whealing responses after short‐term exercise in SD patients. In a previous study, a group of patients underwent low‐, moderate‐and high‐impact exercise, representing 40%, 60%, and 80% of their maximum oxygen uptake, respectively. Cortisol and adrenocorticotropic hormone ACTH levels were higher in the group of SD patients that performed physical activity with moderate and high impact, that is, 60% and 80% maximum oxygen intake after exercise compared to that of their levels before exercise [15]. Given the well‐documented effects of glucocorticoids on mast cell function rather than their general immunosuppressive properties, it is plausible that the observed increase in ACTH and cortisol following provocation testing suppresses mast cell activation and degranulation through direct modulation of key signaling pathways. Specifically, cortisol binding to glucocorticoid receptors (GR) on mast cells is known to inhibit FcεRI expression, reduce Syk phosphorylation, and impair intracellular calcium mobilization, collectively leading to decreased mast cell degranulation [17]. These effects may contribute to an increase in the activation threshold, a reduction in whealing responses, and an overall decrease in SD disease activity and scores.

Our current study found after 1 month of a regular exercise program, whealing responses were lower before and after the exercise program. How exercise affects long‐term disease activity is not known. There also evidence that light and moderate intensity exercise decreases cortisol levels over time and an increase in salivary cortisol output was observed in individuals that undertake vigorous exercise [18].

In another study investigating the relationship between cortisol and exercise, 83 healthy participants were randomly assigned to perform treadmill exercises at light (30% heart rate reserve [HRR]), moderate (50% HRR), and high (70% HRR) intensities. The results demonstrated that as exercise intensity increased, the cortisol response also increased. This finding aligns with our results and may provide insights into the role of anti‐inflammatory effects of cortisol in reducing disease activity in symptomatic dermographism [19].

Our study has several strengths, but also important limitations. One such limitation is the absence of patient‐reported outcome measures (PROMs) to evaluate disease activity and symptom burden from the patients' perspective. While a disease‐specific quality‐of‐life questionnaire for symptomatic dermographism has recently become available [20], there remains no validated PROM specifically tailored to assess disease activity in SD. The inclusion of such tools would have provided more clinically meaningful insights regarding the impact of physical activity on patient well‐being. Additionally, the relatively small sample size limits the generalizability of our findings and highlights the need for future studies involving larger and more diverse patient populations. Another limitation of our study is that we only used the in‐clinic UAS, which reflects the disease activity of the patients’ last day and not for a longer period of time. Furthermore, all patients continued their existing pharmacological treatments (sgAH and/or omalizumab) without dose or frequency modifications during the 1‐month exercise period. Although this ensured intra‐individual comparability, the lack of a washout period may be considered a potential confounding factor when interpreting the results. Lastly, we did not include a separate control group of patients who did not perform any physical activity for 1 month. This decision was based on two main considerations: first, withholding exercise—an intervention known to have general health benefits and recommended by public health authorities—was deemed ethically questionable. Second, we anticipated natural variations in exercise intensity and regularity among patients, which allowed us to perform meaningful subgroup comparisons between those with low and high physical activity levels. Indeed, this design enabled internal contrasts, and our results showed that patients who engaged in higher‐intensity and more consistent exercise experienced greater reductions in whealing responses. Nonetheless, we acknowledge the absence of a non‐exercise control group as a limitation, and future randomized controlled trials are needed to validate our findings and explore causality. In addition, all FricTest measurements were performed by the treating physician in an open‐label fashion, which may introduce observer bias; future studies should incorporate blinded assessors to mitigate this effect.

In summary, our findings demonstrate that SD patients exhibit decreased whealing responses after short‐term exercise and after a long‐term regular exercise program. These findings highlight the importance of regular physical activity and exercise in patients with SD and physicians could recommend regular physical activity as part of the treatment to their SD patients. Identifying additional underlying mechanisms and facilitating factors might improve the understanding of the pathogenesis in CIndU patients.

## Author Contributions

**Ragıp Ertaş:** conceptualization, formal analysis. **Muhammed Burak Yücel:** writing – original draft, project administration, visualization, methodology, resources. **Murat Türk:** methodology, visualization, formal analysis, writing – original draft. **Şule Ketenci Ertaş:** resources. **Emek Kocatürk:** writing – review and editing, visualization. **Melba Muñoz:** writing – review and editing, project administration.

## Conflicts of Interest

R.E. was a speaker and/or advisor for Novartis, Abbvie, Jannsen, and Pfizer. M.B.Y. and Ş.K.E. do not have any conflict of interest to report. M.T. is or recently was a speaker and/or advisor for AstraZeneca, Chiesi, GSK, Novartis, ROXALL, Vem İlaç. E.K. is/was recently a speaker/consultant for Menarini and Novartis and received research funding from Almirall. M.M. is or recently was a speaker and/or advisor for and/or has received research funding from Celltrion, Jasper Therapeutics, Blueprint, Takeda, Biocryst, Annexon, and Roche outside of the submitted manuscript.

## Supporting information

Supporting Information S1

## Data Availability

The data that support the findings of this study are available on request from the corresponding author. The data are not publicly available due to privacy or ethical restrictions.
