# Supplementary material for: Physical Exercise Improves Symptomatic Dermographism
Source: Clin Transl Allergy. 2025 Jul 23;15(7):e70083. doi: 10.1002/clt2.70083 (PMC12286705; doi:10.1002/clt2.70083)
Supplement: Supplementary file 1 — Supporting Information S1 [file CLT2-15-e70083-s001.docx]

**Supplementary Table 1. Individual patient-level mean FricTest® scores before and after short-term (SDE1–SDE2) and one-month (SDE3–SDE4) exercise interventions**

| **Patient Number** | **SDE1** | **SDE2** | **SDE3** | **SDE4** |
| --- | --- | --- | --- | --- |
| **1** | ,58 | 1,00 | 1,25 | ,83 |
| **2** | 2,67 | 2,33 | 2,75 | ,75 |
| **3** | 2,92 | 1,58 | ,75 | ,42 |
| **4** | ,75 | ,25 | ,75 | ,25 |
| **5** | 1,58 | ,00 | 1,42 | 1,42 |
| **6** | 1,17 | ,67 | ,67 | ,58 |
| **7** | ,75 | ,00 | ,92 | ,25 |
| **8** | 3,17 | 2,83 | 3,17 | 3,17 |
| **9** | 3,00 | ,83 | ,67 | ,75 |
| **10** | ,67 | ,00 | ,83 | ,42 |
| **11** | 2,83 | 1,50 | 1,83 | ,58 |
| **12** | 1,92 | 1,17 | 2,58 | 1,58 |
| **13** | 2,58 | ,83 | 1,17 | ,67 |
| **14** | 1,92 | 1,58 | 1,17 | ,58 |
| **15** | 2,08 | 1,42 | 1,58 | ,92 |
| **16** | 2,25 | ,00 | 1,08 | ,67 |
| **17** | 2,17 | ,00 | 1,08 | 1,42 |
| **18** | ,67 | ,00 | ,75 | ,42 |
| **19** | 3,08 | ,00 | 1,25 | ,83 |
| **20** | ,67 | ,00 | 1,92 | ,92 |
| **21** | 2,42 | ,42 | 1,67 | 1,33 |
| **22** | 3,08 | 2,50 | 3,08 | 3,00 |
| **23** | 2,17 | ,00 | 1,00 | ,33 |
| **24** | 1,50 | 1,17 | 2,83 | 2,75 |
| **25** | 2,33 | ,42 | 2,17 | ,50 |
| **26** | 2,25 | ,00 | 1,42 | 1,25 |
| **27** | 2,50 | ,67 | 1,50 | 1,00 |
| **28** | ,83 | ,00 | ,42 | ,42 |
| **29** | 2,25 | ,83 | 2,08 | ,83 |
| **30** | 2,67 | 1,25 | 1,75 | ,67 |
| **31** | ,08 | ,00 | ,58 | ,00 |
| **32** | 2,08 | 1,83 | 1,83 | 1,17 |
| **33** | 2,08 | 1,25 | 3,08 | 3,17 |
| **34** | 2,67 | ,00 | 2,33 | ,67 |

**SDE_1_**: Baseline FricTest score before short-term exercise

**SDE_2_**: Baseline FricTest score after short-term exercise

**SDE_3_**: FricTest score after long-term regular exercise program and before short-term exercise

**SDE_4_**: FricTest score after long-term regular exercise program and after short-term exercise
